# Incremental Value and Cross-Center Validation of Pretreatment Tri-Modal Ultrasound Deep Learning for Predicting Breast Pathologic Complete Response to Neoadjuvant Chemotherapy: A Multicenter Study

**DOI:** 10.3390/cancers18203326

**Published:** 2026-10-15

**Authors:** Chuanmin Wei, Ting Pan, Tiangang Li, Chunyao Zheng, Ying Duan, Fang Nie

**Affiliations:** 1 Ultrasound Medical Center, The Second Hospital of Lanzhou University, Lanzhou 730030, China; yolo_wchuanmin@163.com (C.W.);; 2 Department of Ultrasound Diagnosis, Gansu Provincial Maternity and Child-Care Hospital (Gansu Provincial Central Hospital), Lanzhou 730050, China; 3 Department of Ultrasound, Gansu Provincial Cancer Hospital, Lanzhou 730050, China

**Keywords:** breast cancer, neoadjuvant chemotherapy, pathologic complete response, multimodal ultrasound, deep learning, contrast-enhanced ultrasound

## Abstract

**Objective:** To evaluate the incremental predictive value of pretreatment tri-modal ultrasound deep learning integrating two-dimensional ultrasound (2DUS), color Doppler flow imaging (CDFI), and contrast-enhanced ultrasound (CEUS) for predicting breast pathologic complete response (bpCR) after neoadjuvant chemotherapy (NAC), and to assess performance stability across independent centers. **Methods:** This retrospective multicenter study included 1089 patients with primary invasive breast cancer. Center 1 provided the training cohort (*n* = 572) and internal validation cohort (*n* = 246), whereas centers 2 (*n* = 160) and 3 (*n* = 111) served as independent external validation cohorts. Using pretreatment 2DUS, CDFI, and CEUS within a standardized intratumoral and 10-mm peritumoral framework, single-modality, all three dual-modality combinations, and tri-modal models were developed. The best-performing dual-modality model was identified and locked during training as the primary imaging comparator. A Tri-modal + Clinical model further integrated pretreatment clinical and biomarker variables. Performance was assessed using AUC, Brier score, calibration, decision curve analysis, and ablation experiments. **Results:** Overall, 383 patients (35.2%) achieved bpCR. CEUS was the strongest single modality, and 2DUS + CEUS was the best-performing dual-modality model. Compared with 2DUS + CEUS, the tri-modal model increased the AUC from 0.895 to 0.919 in the combined external cohort (ΔAUC, +0.023; 95% CI, +0.010 to +0.038; *p* = 0.001), with corresponding improvements in Brier score (0.124 to 0.105) and F1 score (0.747 to 0.786). The AUC point estimate increased in both external centers, although the 95% CI for the incremental AUC included zero in external validation cohort 2. Despite modest standalone discrimination, CDFI provided measurable incremental information when added to 2DUS + CEUS. Further integration of pretreatment clinical and biomarker variables increased the combined external AUC to 0.935. Reduced-input retraining showed performance decreases after omission of any modality, with the largest reduction after CEUS omission. **Conclusions:** Pretreatment tri-modal ultrasound deep learning provided incremental predictive value for bpCR beyond the best dual-modality model, with a consistent direction of benefit across two independent external centers. Clinical and biomarker integration provided additional predictive information, supporting further evaluation of this multimodal framework for individualized pretreatment bpCR prediction.

## Simple Summary

Neoadjuvant chemotherapy can eliminate invasive breast cancer in some patients, but response varies substantially and is difficult to predict before treatment. This multicenter study investigated whether combining three pretreatment ultrasound techniques—two-dimensional ultrasound, color Doppler flow imaging, and contrast-enhanced ultrasound—could improve prediction of breast pathologic complete response, defined as no residual invasive cancer in the breast after treatment. We analyzed 1089 patients from three centers and compared single-, dual-, and tri-modal deep learning models. The tri-modal model performed better than the strongest dual-modality model, and adding clinical and biomarker information provided further improvement. The direction of improvement was consistent across two independent external validation centers. These findings suggest that complementary information from multiple ultrasound modalities may provide a noninvasive and more informative approach for pretreatment response prediction in breast cancer.

## 1. Introduction

Neoadjuvant chemotherapy (NAC) is an important treatment strategy for locally advanced and selected high-risk early-stage breast cancer, with the potential to reduce tumor burden, facilitate breast-conserving surgery, and provide an in vivo assessment of tumor sensitivity to systemic therapy [1]. Pathologic complete response is an important post-NAC endpoint and is particularly associated with favorable long-term outcomes in aggressive subtypes such as HER2-positive and triple-negative breast cancer [2,3]. The present study focuses on breast pathologic complete response (bpCR; ypT0/is), defined as the absence of residual invasive carcinoma in the breast irrespective of residual ductal carcinoma in situ. Because response to NAC reflects a combination of tumor burden, molecular characteristics, and the local tumor microenvironment, reliable pretreatment identification of patients likely to achieve bpCR remains challenging.

Ultrasound provides complementary structural and vascular information without ionizing radiation and is widely available in routine breast imaging. 2DUS primarily depicts tumor morphology and internal echotexture, CDFI characterizes macroscopic vascularity and blood-flow patterns, and CEUS provides information on microvascular enhancement and perfusion heterogeneity [4,5]. In addition, treatment-response-related imaging phenotypes may extend beyond the tumor itself. Peritumoral tissue, including vascular, stromal, and tumor–host interface components, may contain complementary predictive information [6,7]. These considerations provide a rationale for integrating intratumoral and adjacent peritumoral information across multiple ultrasound modalities.

Ultrasound-based deep learning, radiomics, and multimodal fusion have increasingly been investigated for predicting pathologic response to NAC [8,9,10]. However, most studies have emphasized the overall performance of a final combined model relative to single-modality or clinical models. In a tri-modal setting, showing that a tri-modal model outperforms an individual modality does not establish whether the third modality contributes meaningful information beyond an already strong dual-modality baseline. A more rigorous assessment therefore requires identifying the best-performing dual-modality model during development and subsequently testing whether addition of the third modality provides incremental improvement in discrimination and probabilistic performance. Furthermore, because ultrasound is sensitive to imaging platforms, acquisition settings, and operator experience, whether such incremental benefit persists across independent centers is important for evaluating potential transportability.

To address these gaps, we developed single-modality, all three dual-modality combinations, and tri-modal deep learning models using pretreatment 2DUS, CDFI, and CEUS within a standardized intratumoral–peritumoral framework. The primary objective was to quantify the incremental predictive value of tri-modal fusion over the best-performing dual-modality model identified and locked during training, and to evaluate the consistency of this incremental benefit across two independent external validation cohorts. We further assessed the additional value of pretreatment clinical and biomarker variables beyond the complete tri-modal imaging representation. Calibration, decision curve analysis, ablation experiments, and gradient-weighted class activation mapping (Grad-CAM) were used to evaluate probabilistic performance, potential clinical utility, component contributions, and model interpretability.

## 2. Materials and Methods

### 2.1. Study Design and Patient Cohorts

This retrospective multicenter study consecutively enrolled patients with primary invasive breast cancer who underwent NAC, followed by breast surgery and postoperative pathologic assessment, at the Second Hospital of Lanzhou University, Gansu Provincial Cancer Hospital, and Gansu Provincial Maternity and Child-care Hospital between January 2020 and January 2026. The study design and reporting were guided by the relevant recommendations of TRIPOD + AI and CLAIM 2024 for clinical prediction models and artificial intelligence research in medical imaging [11,12].

Patients were eligible if they met all of the following criteria: (1) biopsy-proven primary invasive breast cancer; (2) completion of standard NAC followed by breast surgery; (3) availability of pretreatment 2DUS, CDFI, and CEUS, with image quality sufficient for target lesion identification and intratumoral–peritumoral region analysis; (4) complete pretreatment data on age, maximum tumor diameter, clinical tumor stage (cT), clinical nodal stage (cN), estrogen receptor (ER), progesterone receptor (PR), human epidermal growth factor receptor 2 (HER2), and Ki-67; and (5) complete postoperative breast pathology allowing definitive determination of bpCR status. Exclusion criteria were as follows: receipt of breast cancer-related treatment before NAC that could alter baseline imaging phenotypes; concurrent active malignancy; bilateral breast cancer or ipsilateral multicentric/multifocal disease; inability to reliably match the target lesion with postoperative pathology; inadequate 2DUS, CDFI, or CEUS image quality for region construction and model analysis; inability to reproducibly construct the peritumoral region; or missing key clinical, pathologic, or outcome data. All consecutive patients who met these criteria during the study period were included in the analysis.

A total of 1089 patients were included. At center 1, 818 patients were randomly divided at the patient level into a training cohort (n = 572) and an internal validation cohort (n = 246). Patients from center 2 (n = 160) and center 3 (n = 111) constituted external validation cohorts 1 and 2, respectively. The two external validation cohorts were additionally pooled to form a combined external validation cohort (n = 271) for an overall assessment of external model performance. All 2DUS, CDFI, and CEUS images and their derived regions from the same patient were assigned to the same dataset to prevent within-patient information leakage. The training cohort was used for model parameter estimation, cross-validation, and model selection, whereas the internal validation cohort was used for independent evaluation of model performance within the development center. Neither external validation cohort was involved in parameter estimation, hyperparameter selection, classification-threshold determination, or center-specific recalibration. Patient selection, cohort allocation, and the overall study workflow are illustrated in Figure 1.

The study was conducted in accordance with the Declaration of Helsinki and was approved by the institutional ethics committees of all participating centers, including the Medical Ethics Committee of The Second Hospital of Lanzhou University (protocol code 2024A-499; approval date: 21 March 2024).

### 2.2. Clinical and Biomarker Data and Reference Standard

Pretreatment clinical and biomarker data were retrospectively retrieved from the electronic medical records, imaging records, and pathology systems at each center. Variables used for modeling included age, pretreatment maximum tumor diameter, cT, cN, ER, PR, HER2, and Ki-67. Uniform variable definitions and coding rules were applied across centers. Continuous variables were retained at their original values, with standardization parameters estimated exclusively from the training cohort and then fixed for all validation cohorts. Because complete data for all core predictors were required for inclusion, no imputation was performed in the primary analysis.

ER and PR positivity was defined as nuclear staining in ≥1% of tumor cells on pretreatment core-needle biopsy. HER2 status was determined by immunohistochemistry, with in situ hybridization used for equivocal IHC 2+ cases. Ki-67 was recorded as the percentage of positively stained tumor cells and entered into the model as a continuous variable. Hormone receptor (HR) positivity was defined as ER and/or PR positivity. Molecular subtypes were operationally categorized using pretreatment surrogate clinicopathologic criteria: Luminal A, HR-positive/HER2-negative with Ki-67 < 20%; Luminal B, HR-positive/HER2-negative with Ki-67 ≥ 20% or HR-positive/HER2-positive; HER2-positive non-luminal, HR-negative/HER2-positive; and TNBC, ER/PR/HER2-negative [13,14]. Molecular subtype was used only for baseline characterization and exploratory subgroup analyses and was not entered as a separate model predictor.

All patients completed NAC, whereas specific treatment regimens were not included as model predictors. The primary outcome was bpCR, defined as the absence of residual invasive carcinoma in the breast after NAC (ypT0/is), irrespective of residual ductal carcinoma in situ; any residual invasive carcinoma was classified as non-bpCR. Post-treatment axillary nodal status was not included in the bpCR definition. Surgical specimens were evaluated according to routine breast pathology procedures by two experienced breast pathologists blinded to model predictions. Disagreements were resolved by consensus, and the consensus postoperative pathologic assessment served as the reference standard.

### 2.3. Multimodal Ultrasound Examination and Image Acquisition

All ultrasound examinations were performed before NAC by physicians experienced in breast ultrasonography. Standardized principles for lesion visualization and image quality control were applied across centers, including complete visualization of the target lesion, appropriate adjustment of imaging depth and focal position, and acquisition of the same or most comparable lesion plane across modalities whenever feasible.

For 2DUS, lesions were assessed in multiple planes, and representative images showing the maximum tumor extent and internal characteristics were retained. CDFI was acquired on the same or a closely matched lesion plane, with color gain, pulse repetition frequency, and wall-filter settings adjusted to optimize visualization of intratumoral and adjacent blood-flow signals while minimizing artifacts. Original color-flow information was preserved for model analysis.

CEUS was performed using a low-mechanical-index contrast-specific mode after intravenous administration of SonoVue^®^ (Bracco Imaging S.p.A., Colleretto Giacosa, Italy; 0.02 mL/kg), followed by a 5-mL saline flush. The mechanical index was maintained at 0.08–0.10, and dynamic imaging was recorded for 60 s at approximately 15 frames/s. A representative peak-enhancement frame with clear lesion boundaries and minimal motion artifact was selected for deep learning analysis. Thus, CEUS primarily represented peak-phase microvascular enhancement and perfusion heterogeneity rather than full temporal perfusion kinetics. This single-frame representation was used to maintain a standardized two-dimensional input format across the three ultrasound modalities. All images were digitally exported, de-identified, and subjected to standardized quality review before model analysis.

### 2.4. Image Preprocessing, ROI Construction, and Quality Control

A standardized preprocessing pipeline was applied to all modalities. After de-identification and removal of measurement annotations and irrelevant interface elements, images were cropped around the target lesion and resized to 224 × 224 pixels. Grayscale 2DUS images were replicated across three channels, whereas the original RGB information was retained for CDFI and CEUS. Pixel intensities were first scaled to the range of 0–1 and then normalized using the ImageNet mean (0.485, 0.456, 0.406) and standard deviation (0.229, 0.224, 0.225), consistent with the ImageNet-pretrained ResNet-50 backbone. Online data augmentation was applied only to the training cohort and included random horizontal flipping (probability, 0.5), rotation within ±10°, translation of up to 5% of the image width or height, and isotropic scaling within 0.95–1.05. Vertical flipping, color-intensity perturbation, and elastic deformation were not used to avoid altering anatomically or hemodynamically relevant imaging characteristics. No data augmentation was applied to the internal or external validation cohorts.

The ITS was manually delineated along the visible tumor boundary on each modality. ROI delineation was performed without access to postoperative pathologic response or model predictions. 2DUS served as the primary anatomic reference, while CDFI and CEUS were matched at the patient level using lesion orientation, depth, contour, and adjacent anatomic landmarks. Boundaries were independently reviewed and adjusted according to modality-specific lesion visibility, thereby maintaining correspondence to the same target lesion without requiring strict pixel-level cross-modal registration.

A 10-mm PTS was generated by outward expansion from the tumor boundary, excluding the ITS. The 10-mm width was used to standardize representation of the immediately adjacent peritumoral region while limiting inclusion of more distant breast tissue. Portions extending beyond the breast tissue into the skin, chest wall, image boundary, or clearly non-breast structures were clipped according to anatomic boundaries. The same 10-mm PTS definition was applied to all modalities and cohorts without re-optimization in validation data. Each patient therefore contributed six region-level inputs (ITS and PTS for 2DUS, CDFI, and CEUS). For reproducibility assessment, 109 randomly selected patients underwent independent repeat delineation by a second blinded physician, and interobserver agreement was quantified using the Dice similarity coefficient. For these 109 patients, the two independently delineated ROI sets were additionally evaluated using the locked Tri-modal model to assess the robustness of patient-level predicted probabilities and model discrimination to interobserver ROI variation; agreement was quantified using the intraclass correlation coefficient (ICC) and mean absolute probability difference, and the corresponding AUCs were compared descriptively.

### 2.5. Model Architecture and Hierarchical Multimodal Fusion

To enable standardized comparison across ultrasound modalities and their combinations, all imaging models used the same feature-encoding, regional-fusion, and classification framework, differing only in the input modality combination (Figure 2). For each ultrasound modality, an ImageNet-pretrained ResNet-50 was used as the feature encoder. The ITS and PTS within the same modality shared encoder parameters, and convolutional feature extraction followed by global average pooling was used to obtain fixed-dimensional region-level representations.

A learnable region-attention mechanism was applied to adaptively integrate the ITS and PTS features within each ultrasound modality. For the m-th modality, the patient-level modality representation was defined as

$$f_{m}=\alpha_{m,ITS}f_{m,ITS}+\alpha_{m,PTS}f_{m,PTS}$$

where $f_{m,ITS}$ and $f_{m,PTS}$ denote the intratumoral and peritumoral feature representations, respectively, and $\alpha_{m,ITS}$ and $\alpha_{m,PTS}$ are learnable normalized attention weights satisfying

$$\alpha_{m,ITS}+\alpha_{m,PTS}=1.$$

For dual-modality and tri-modal models, modality-specific representations were further integrated using a learnable modality-attention mechanism:

$$f_{multi}=\sum_{m=1}^{M}\beta_{m}f_{m}$$

where M denotes the number of modalities included in the model, $f_{m}$ is the modality-specific representation obtained after region-attention fusion, and $\beta_{m}$ is the corresponding learnable normalized modality-attention weight, satisfying

$$\sum_{m=1}^{M}\beta_{m}=1$$

Here, M = 2 for dual-modality models and M = 3 for the tri-modal model. This mechanism enabled adaptive weighting of the participating ultrasound modalities during feature fusion. The fused tri-modal representation was passed through a fully connected classification layer with a sigmoid activation function to generate the patient-level probability of bpCR. All single-modality, dual-modality, and tri-modal models used the same encoder architecture, regional-fusion strategy, and classification framework so that differences in predictive performance primarily reflected differences in the imaging information provided by the input modality combinations.

As a conventional pretreatment reference model, a Clinical/Biomarker model was constructed using L2-regularized logistic regression with age, pretreatment maximum tumor diameter, cT, cN, ER, PR, HER2, and Ki-67. A Tri-modal + Clinical model (integrating clinical and biomarker variables) was further developed by mapping these variables through a fully connected layer to obtain a structured clinical representation, which was then fused with the tri-modal imaging representation to generate the final bpCR probability.

The primary imaging comparison contrasted the best-performing dual-modality model identified and locked during training with the tri-modal model, thereby quantifying the incremental predictive value of adding the third ultrasound modality beyond the strongest dual-modality baseline. The comparison between the tri-modal and Tri-modal + Clinical models was considered a secondary analysis to assess the additional value of pretreatment clinical and biomarker information beyond the complete tri-modal imaging representation.

**Figure 2:**
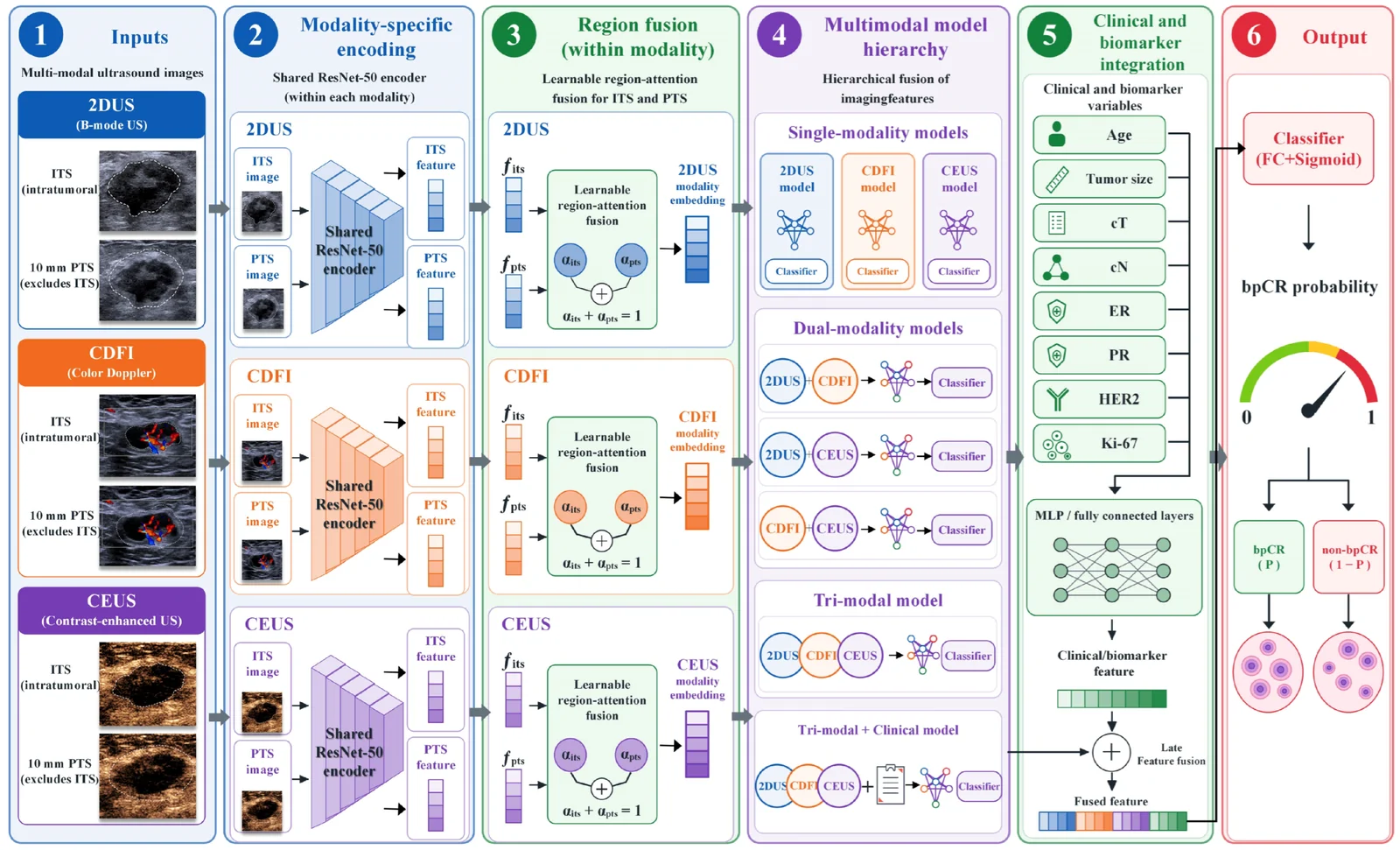
Hierarchicaltri-modal ultrasound deep learning architecture integrating modality-specific feature extraction, intratumoral–peritumoral region fusion, multimodal fusion, and pretreatment clinical and biomarker information. Blue, orange, purple, and green denote 2DUS, CDFI, CEUS, and clinical/biomarker components, respectively; arrows indicate the direction of feature flow and fusion.

### 2.6. Model Development and Independent Validation

All model development, model selection, and hyperparameter tuning were restricted to the training cohort. Stratified five-fold cross-validation was performed at the patient level, with the proportion of bpCR and non-bpCR cases preserved across folds. Imaging models were optimized using weighted binary cross-entropy loss and the AdamW optimizer (initial learning rate, 1 × 10^−4^; weight decay, 1 × 10^−4^; batch size, 16). Class weights were determined from the outcome distribution in the training cohort to reduce the influence of class imbalance. The learning rate was adjusted using cosine annealing from the initial learning rate to a minimum of 1 × 10^−6^. Models were trained for a maximum of 100 epochs, with early stopping applied when the validation loss failed to improve for 15 consecutive epochs. Identical cross-validation partitions, preprocessing and augmentation strategies, hyperparameters, and a fixed random seed were used across all candidate imaging models to ensure consistent model comparison. As a sensitivity analysis, the primary 2DUS + CEUS versus Tri-modal comparison was additionally repeated across five random seeds while keeping the data partitions and all other training settings unchanged.

Training-stage out-of-fold (OOF) predictions were used to compare the three dual-modality models. The dual-modality model with the highest OOF AUC was identified as the primary imaging comparator and locked before evaluation on the internal and external validation cohorts. After model selection, the network architecture, preprocessing pipeline, hyperparameters, and training strategy were fixed. Final models were retrained using the complete training cohort, with the number of training epochs determined from the cross-validation results without reference to any independent validation cohort. Classification thresholds were determined from training-stage OOF probabilities using the Youden index and subsequently fixed for all validation cohorts.

The internal validation cohort and the two external validation cohorts were used exclusively for independent model evaluation and did not contribute to feature learning, model selection, hyperparameter tuning, classification-threshold determination, or center-specific recalibration. All final models were applied to the external cohorts without retraining, fine-tuning, parameter updating, or threshold adjustment.

### 2.7. Ablation and Interpretability Analyses

Three ablation settings were used to assess the contributions of imaging modalities, spatial regions, and fusion mechanisms. Reduced-input retraining analyses omitted 2DUS, CDFI, or CEUS individually and retrained the corresponding models using the same training and evaluation procedures; spatial ablation retained either ITS or PTS alone; and fusion ablation replaced region-attention with direct ITS–PTS feature concatenation and modality-attention with direct concatenation of modality-specific representations. All ablation models followed the same training and evaluation procedures as the full tri-modal model, with performance reported across the validation cohorts and the combined external cohort used for the primary interpretation of ablation effects.

To further assess whether the incremental performance of the Tri-modal model could be explained by the additional encoder capacity, an architecture-matched three-encoder control was additionally trained by replacing the CDFI input with a duplicated 2DUS input, while retaining the same three-encoder architecture and keeping all other preprocessing, training partitions, hyperparameters, and optimization procedures unchanged.

Model interpretability was evaluated using Grad-CAM [15]. Activation maps from each modality-specific encoder were overlaid on the corresponding ultrasound images together with ITS and 10-mm PTS boundaries. Representative bpCR and non-bpCR cases were visualized to illustrate modality-specific spatial attention. Grad-CAM was used only to characterize prediction-related attention and was not interpreted as evidence of histopathologic invasion or biological causality.

### 2.8. Statistical Analysis

Continuous variables were summarized as mean ± standard deviation or median (interquartile range), and categorical variables as counts and percentages. Between-cohort comparisons were performed using analysis of variance or the Kruskal–Wallis test for continuous variables and the χ^2^ test or Fisher’s exact test for categorical variables, as appropriate; corresponding two-group tests were used for bpCR versus non-bpCR comparisons. All tests were two-sided, with *p* < 0.05 considered statistically significant.

Model performance was evaluated using AUC with 95% confidence intervals (CIs), accuracy, sensitivity, specificity, PPV, NPV, F1 score, and Brier score. Classification metrics were calculated using thresholds determined exclusively from the training cohort. AUCs and their 95% CIs were estimated using the DeLong method, and paired AUCs were compared using the DeLong test. Ninety-five percent CIs for ΔAUC, ΔAccuracy, ΔF1, and ΔBrier were estimated using patient-level paired bootstrap resampling with 2000 iterations. Calibration was assessed using calibration curves, intercepts, and slopes. Decision curve analysis was performed over threshold probabilities of 0.10–0.60 to evaluate net benefit. Relative Brier reduction was calculated as [(Brier_ref − Brier_new)/Brier_ref] × 100%.

Exploratory subgroup analyses were performed in the combined external cohort according to age (≤50 vs. >50 years), tumor size (≤30 vs. >30 mm), cT stage (cT1–2 vs. cT3–4), cN stage (cN0–1 vs. cN2–3), molecular subtype, ER status, HER2 status, and Ki-67 index (≤30% vs. >30%). Subgroup-specific AUCs with 95% CIs were reported for the Tri-modal model. Heterogeneity in the association between the continuous Tri-modal prediction score and bpCR across subgroup levels was assessed using score-by-subgroup interaction terms in logistic regression; *p*-values for interaction were obtained using likelihood-ratio tests, with multi-level interactions tested jointly. All subgroup analyses were considered exploratory, and no adjustment for multiple comparisons was applied. ROI delineation reproducibility was assessed using the Dice similarity coefficient. For the repeat-delineation subset, prediction-level agreement was further assessed using the intraclass correlation coefficient and mean absolute probability difference, with corresponding AUCs compared descriptively. Statistical analyses were performed using R version 4.4.1 and Python version 3.11.

## 3. Results

### 3.1. Patient Characteristics, bpCR Distribution, and ROI Reproducibility

A total of 1089 patients were included, comprising 572 in the training cohort, 246 in the internal validation cohort, 160 in external validation cohort 1, and 111 in external validation cohort 2. Overall, 383 patients (35.2%) achieved bpCR, whereas 706 (64.8%) were classified as non-bpCR. The bpCR rates were 35.1%, 36.2%, 36.3%, and 31.5% across the four cohorts, respectively, with no significant between-cohort difference (*p* = 0.841). Among the major clinicopathologic characteristics, only the distribution of cT stage differed significantly across centers (*p* = 0.041), whereas no significant between-cohort differences were observed for the remaining characteristics (all *p* > 0.05). The proportion of patients with cT3 disease was higher in external validation cohort 2 (27.0%) than in the other cohorts (14.0–17.5%) (Table 1).

In the overall cohort, 462 (42.4%), 313 (28.7%), 163 (15.0%), and 151 (13.9%) patients had Luminal A, Luminal B, HER2-positive non-luminal, and triple-negative breast cancer (TNBC), respectively, with corresponding bpCR rates of 17.7%, 34.5%, 63.8%, and 58.9%. Compared with patients with non-bpCR, those who achieved bpCR had a smaller pretreatment maximum tumor diameter [33.4 (26.6–40.9) mm vs. 41.4 (33.7–49.5) mm], a higher Ki-67 index [48.0% (28.0–68.0) vs. 32.0% (12.0–52.0)], and generally lower cT and cN stages. The bpCR group also had lower proportions of ER-positive (49.6% vs. 82.9%) and PR-positive (44.4% vs. 69.3%) tumors but a higher proportion of HER2-positive tumors (33.9% vs. 14.3%) (all *p* < 0.001). Age did not differ significantly between the bpCR and non-bpCR groups (Supplementary Table S1).

Among the 109 patients who underwent repeat ROI delineation, the Dice similarity coefficients were 0.923 ± 0.019 for 2DUS, 0.897 ± 0.027 for CDFI, and 0.912 ± 0.023 for CEUS, indicating high spatial reproducibility of ROI delineation across all three modalities. Prediction-level analysis further showed high agreement between the two independently delineated ROI sets, with an ICC of 0.946 and a mean absolute probability difference of 0.034. The corresponding AUCs were 0.913 and 0.905 for the primary and repeat ROI sets, respectively, with an absolute AUC difference of 0.008, suggesting that interobserver ROI variation had only a minimal impact on model discrimination (Supplementary Table S2).

### 3.2. Performance Hierarchy of Single- and Dual-Modality Models

The performance ranking observed during model development was generally consistent with that in the subsequent independent validation cohorts. Training-stage OOF evaluation identified 2DUS + CEUS as the best-performing dual-modality model (AUC, 0.913), compared with 2DUS + CDFI (0.883) and CDFI + CEUS (0.894); it was therefore locked as the primary imaging comparator (Supplementary Table S3).

In the combined external validation cohort, the AUCs of the Clinical/Biomarker, 2DUS, CDFI, and CEUS models were 0.805, 0.823, 0.765, and 0.852, with corresponding Brier scores of 0.171, 0.162, 0.184, and 0.149, respectively. Among the dual-modality models, the AUCs were 0.864 for 2DUS + CDFI, 0.895 for 2DUS + CEUS, and 0.879 for CDFI + CEUS, identifying CEUS and 2DUS + CEUS as the best-performing single- and dual-modality models, respectively (Table 2). Overall, dual-modality fusion yielded higher discrimination than the corresponding individual modalities, with 2DUS + CEUS showing consistently strong performance across the validation cohorts.

The locked 2DUS + CEUS model achieved AUCs of 0.904, 0.901, 0.887, and 0.895 in the internal validation, external validation cohort 1, external validation cohort 2, and combined external validation cohort, respectively. In the combined external cohort, its accuracy, sensitivity, specificity, PPV, NPV, and F1 score were 81.5%, 79.6%, 82.6%, 70.5%, 88.6%, and 0.747, respectively, with a Brier score of 0.124 (Table 3).

### 3.3. Incremental Value of Tri-Modal Fusion over the Best Dual-Modality Model

Adding CDFI to 2DUS + CEUS yielded higher AUCs across all validation cohorts. The tri-modal model achieved AUCs of 0.928, 0.925, 0.911, and 0.919 in the internal validation, external validation cohort 1, external validation cohort 2, and combined external validation cohort, respectively, representing consistent AUC improvements across all validation cohorts. In the combined external cohort, the ΔAUC was +0.023 (95% CI, +0.010 to +0.038; *p* = 0.001) (Table 4; Figure 3).

Paired DeLong tests showed that the AUC improvement was statistically significant in the internal validation cohort (*p* = 0.002), external validation cohort 1 (*p* = 0.011), and combined external validation cohort (*p* = 0.001). In external validation cohort 2, the point estimate for ΔAUC was +0.023, but the 95% CI included zero (−0.004 to +0.052; *p* = 0.084). Across five random seeds, the mean AUCs of the 2DUS + CEUS and Tri-modal models in the combined external cohort were 0.895 ± 0.002 and 0.918 ± 0.004, respectively. The mean ΔAUC was +0.023 ± 0.003 (range, +0.019 to +0.025), with the incremental improvement remaining positive across all seeds.

In the combined external cohort, the tri-modal model increased accuracy from 81.5% to 84.5% and the F1 score from 0.747 to 0.786, corresponding to a ΔAccuracy of +0.030 (95% CI, +0.009 to +0.052) and a ΔF1 of +0.039 (95% CI, +0.013 to +0.074). The Brier score decreased from 0.124 to 0.105, with a ΔBrier of −0.019, corresponding to a 15.3% relative reduction. Although CDFI alone showed relatively modest discrimination in the combined external cohort (AUC, 0.765), its addition to 2DUS + CEUS increased the AUC from 0.895 to 0.919. Consistently, the reduced-input model retrained without CDFI achieved a combined external AUC of 0.895 (Supplementary Table S4).

### 3.4. Additional Value of Clinical and Biomarker Variables

The Clinical/Biomarker model achieved an AUC of 0.805 in the combined external validation cohort, lower than that of the primary imaging-fusion models. After integrating age, pretreatment maximum tumor diameter, cT, cN, ER, PR, HER2, and Ki-67 with the tri-modal imaging representation, the Tri-modal + Clinical model achieved AUCs of 0.942, 0.939, 0.927, and 0.935 in the internal validation, external validation cohort 1, external validation cohort 2, and combined external validation cohort, respectively.

In the combined external cohort, the Tri-modal + Clinical model yielded a ΔAUC of +0.016 over the tri-modal model (95% CI, +0.008 to +0.024; *p* = 0.002). The Brier score decreased from 0.105 to 0.094, while accuracy increased from 84.5% to 86.3% and the F1 score from 0.786 to 0.810. Sensitivity, specificity, PPV, and NPV were 84.9%, 87.1%, 77.5%, and 91.7%, respectively.

The incremental AUC improvement from the tri-modal to the Tri-modal + Clinical model in the combined external cohort (+0.016) was numerically smaller than the primary imaging increment from the best dual-modality to the tri-modal model (+0.023). The additional ΔAUC was +0.014 in external validation cohort 1 (95% CI, +0.004 to +0.025; *p* = 0.018) and +0.016 in external validation cohort 2 (95% CI, −0.001 to +0.034; *p* = 0.069). Thus, the direction of improvement remained positive in both external cohorts, although statistical significance was not reached in external validation cohort 2. Overall, clinical and biomarker variables provided additional, albeit more modest, predictive value beyond the tri-modal imaging representation.

### 3.5. Cross-Center Performance Stability, Calibration, and Potential Clinical Utility

The tri-modal and Tri-modal + Clinical models maintained relatively stable discrimination and probabilistic performance across the two independent external centers. Relative to internal validation, the AUCs of the tri-modal model were lower by 0.003 and 0.017 in external validation cohorts 1 and 2, respectively, while the corresponding differences for the Tri-modal + Clinical model were −0.003 and −0.015. In the combined external cohort, the corresponding differences were −0.009 and −0.007, respectively. Brier scores also showed only modest variation across validation cohorts, with maximum increases of no more than 0.009 for the tri-modal model and 0.008 for the Tri-modal + Clinical model (Table 5). All external evaluations used the models and classification thresholds locked during training, without retraining, threshold optimization, or center-specific recalibration.

External validation cohort 2 had a higher proportion of patients with cT3 disease (27.0%) and a lower bpCR rate (31.5%). In this cohort, the tri-modal model achieved an AUC of 0.911, a Brier score of 0.110, and a calibration slope of 0.990, while the corresponding values for the Tri-modal + Clinical model were 0.927, 0.097, and 1.000, respectively. The ΔAUC from the best dual-modality to the tri-modal model remained +0.023, consistent in direction with the other validation cohorts, although its 95% CI was wider and crossed zero. Overall, both models maintained relatively stable discrimination, probabilistic performance, and calibration across cohorts with differing patient characteristics.

Calibration and decision curve analyses for the combined external validation cohort are shown in Figure 4. Across the evaluated threshold probability range of 0.10–0.60, the tri-modal and Tri-modal + Clinical models generally provided greater net benefit than the best dual-modality model and outperformed the treat-all and treat-none strategies across most evaluated thresholds. At a threshold probability of 0.50, the net benefits were 0.153, 0.203, and 0.228 for the best dual-modality, tri-modal, and Tri-modal + Clinical models, respectively (Supplementary Table S5). Exploratory subgroup AUCs ranged from 0.902 to 0.936, with no statistically significant score-by-subgroup interactions (all *p* for interaction > 0.05) (Figure 5B; Supplementary Table S6). In molecular-subtype analyses, the incremental AUC over the best dual-modality model remained positive across all four subtypes (+0.023 to +0.036; Supplementary Table S7).

### 3.6. Ablation Experiments and Model Interpretability

The full tri-modal model achieved an AUC of 0.919 in the combined external validation cohort. The architecture-matched three-encoder control achieved AUCs of 0.906, 0.903, 0.889, and 0.898 in the internal, external 1, external 2, and combined external cohorts, respectively. Its combined external AUC was only slightly higher than that of 2DUS + CEUS (0.898 vs. 0.895) and remained lower than that of the Tri-modal model (0.919), suggesting that increased model capacity alone was insufficient to reproduce the performance gain observed with the true CDFI input. In reduced-input retraining analyses, models trained without CEUS, 2DUS, or CDFI achieved combined external AUCs of 0.864, 0.879, and 0.895, respectively. When only the ITS or PTS was retained, the AUC decreased to 0.901 and 0.882, respectively. Removing the region-attention mechanism and directly concatenating ITS and PTS features reduced the AUC to 0.907, whereas removing the modality-attention mechanism and directly concatenating modality-specific representations reduced the AUC to 0.910. Reduced-input retraining, spatial-region ablation, and fusion ablation were consistently associated with lower performance, with the largest decrease observed for the model retrained without CEUS (Table 6; Figure 5A).

Grad-CAM results are shown in Figure 6. In representative bpCR and non-bpCR cases, the three ultrasound modalities exhibited distinct spatial attention patterns. High-activation regions on 2DUS were predominantly located within the tumor, along the tumor boundary, and in portions of the adjacent peritumoral region. CDFI attention was more concentrated in regions associated with intratumoral and peritumoral color-flow signals, whereas CEUS attention predominantly involved areas of pronounced enhancement and the tumor–peritumoral interface. High-activation regions showed incomplete overlap across the three modalities. Grad-CAM was used solely to visualize prediction-related spatial attention and should not be interpreted as delineating histopathologic invasion, establishing biological causality, or identifying mechanisms of treatment response.

## 4. Discussion

In this multicenter study of 1089 patients with breast cancer, pretreatment tri-modal deep learning integrating 2DUS, CDFI, and CEUS provided incremental predictive value for bpCR beyond the best-performing dual-modality model. A key feature of the study design was the systematic evaluation of all single- and dual-modality combinations, with the best dual-modality model identified and locked during training as the primary imaging comparator. CEUS was the strongest single modality, and 2DUS + CEUS was the strongest dual-modality combination; adding CDFI yielded higher AUCs across the internal and both external validation cohorts. The direction of this incremental benefit was consistent across the two external centers, although statistical significance was not reached in external validation cohort 2. Further integration of pretreatment clinical and biomarker variables yielded an additional, albeit smaller, improvement. Together, these findings support complementary predictive contributions of the three ultrasound modalities beyond a strong dual-modality imaging baseline.

The strong performance of CEUS and the 2DUS + CEUS combination may reflect complementary structural and microvascular information. 2DUS primarily characterizes tumor morphology, margins, and internal echotexture, whereas CEUS reflects microvascular enhancement and perfusion-related heterogeneity. Previous studies have demonstrated the predictive potential of conventional ultrasound, blood-flow imaging, and CEUS-derived features for pathologic response to NAC [16,17,18,19,20], with multimodal approaches providing additional predictive information. Importantly, all modality combinations in the present study were evaluated within the same spatial and network framework, with the best-performing dual-modality model selected exclusively during training and locked as the primary imaging comparator. This design allowed the incremental value of the third modality to be assessed against a strong dual-modality baseline rather than against individual modalities alone. Because CEUS input was limited to a representative peak-enhancement frame, its contribution should be interpreted as reflecting peak-phase microvascular enhancement rather than full temporal perfusion kinetics. Future studies incorporating dynamic CEUS information may determine whether temporal perfusion kinetics provide additional predictive value beyond peak-phase spatial enhancement.

The CDFI findings highlight that standalone discrimination and incremental contribution are not equivalent. Although CDFI showed relatively modest standalone performance, adding it to 2DUS + CEUS was associated with higher discrimination and better probabilistic performance, while the model retrained without CDFI showed lower performance. This may reflect differences in the vascular information captured by CDFI and CEUS: CDFI primarily characterizes larger-vessel distribution and macroscopic blood flow, whereas CEUS reflects microvascular enhancement and tissue perfusion [21,22]. Therefore, the value of an imaging modality within a multimodal framework should be assessed not only by its standalone performance but also by the additional information it contributes beyond an established model. Consistently, the architecture-matched control achieved a combined external AUC of 0.898, compared with 0.919 for the true Tri-modal model, indicating that increased model capacity alone did not reproduce the performance gain observed with the true CDFI input.

Spatial ablation supported complementary predictive information from both intratumoral and adjacent peritumoral regions, as performance decreased when either region was used alone or when region-attention fusion was removed. Previous studies have suggested that peritumoral tissue may contain imaging phenotypes related to the tumor–host interface, stromal alterations, and vascular environment [23,24,25]. In this study, the standardized 10-mm PTS enabled intratumoral and adjacent peritumoral information to be represented within a unified framework. Importantly, this 10-mm range was an imaging observation region rather than a histopathologic invasion distance, pathologic margin, or surgical safety margin.

Clinical and biomarker variables provided additional information beyond the complete tri-modal imaging representation, consistent with previous multimodal studies reporting complementary value from combining imaging and clinical information [26,27]. Their integration increased the combined external AUC from 0.919 to 0.935 while further reducing the Brier score. However, the corresponding ΔAUC (+0.016) was numerically smaller than the primary imaging increment from the best dual-modality to the tri-modal model, suggesting a complementary but comparatively modest contribution beyond the complete imaging representation.

Cross-center stability is important for the potential transportability of ultrasound-based models because image characteristics may vary across equipment, acquisition settings, operators, and patient populations [28,29]. In this study, both external centers were evaluated using fully locked models without retraining, threshold optimization, or center-specific recalibration. Only modest decreases in AUC were observed relative to internal validation, with similarly stable Brier scores and calibration slopes. Notably, external validation cohort 2 had a higher proportion of cT3 disease and a lower bpCR rate, yet the tri-modal model retained an AUC of 0.911 and a calibration slope close to 1.0. Although the incremental AUC did not reach statistical significance in this cohort, its direction remained consistent. These findings support cross-center performance stability within the present multicenter setting, while emphasizing that external performance should be interpreted jointly from effect estimates, confidence intervals, discrimination, calibration, and probabilistic performance.

Beyond discrimination, calibration and decision-analytic performance are relevant to potential clinical use [30]. In the combined external cohort, the tri-modal and Tri-modal + Clinical models showed favorable Brier scores and calibration slopes close to 1.0. DCA also indicated generally greater net benefit than the best dual-modality model across the evaluated threshold range. However, DCA represents model-based estimates of net benefit and does not replace prospective evaluation of clinical impact. For patients predicted to have a low probability of bpCR, such pretreatment risk stratification may also support future evaluation of adaptive or alternative treatment strategies, including emerging investigational physical and bioelectronic approaches [31].

Grad-CAM provided complementary visualization of modality-specific spatial attention. High-activation regions differed across 2DUS, CDFI, and CEUS, consistent with the component-analysis findings and supporting complementary predictive contributions across modalities [32]. Exploratory subgroup analyses did not show clear evidence of marked performance heterogeneity across the evaluated clinical and molecular strata, while the incremental AUC over the best dual-modality model remained positive across all four molecular subtypes. However, several subgroup strata were relatively small, and the absence of statistically significant interaction should not be interpreted as evidence of equivalent model performance across subgroups. Grad-CAM likewise reflects prediction-related attention rather than histopathologic invasion or biological causality.

Several limitations should be acknowledged. First, the retrospective design and requirement for complete pretreatment 2DUS, CDFI, and CEUS data may have introduced selection bias. Second, differences in ultrasound equipment and operator-dependent acquisition may have contributed to image heterogeneity despite standardized acquisition and quality-control principles. Third, the three modalities were matched at the lesion level rather than by strict pixel-level registration. Fourth, ROI delineation was performed manually. Although interobserver reproducibility was high, manual delineation may limit scalability and workflow efficiency in future clinical implementation. Fifth, CEUS analysis used a representative peak-enhancement frame and did not exploit the full temporal information of the dynamic sequence. Sixth, treatment-regimen heterogeneity was not specifically modeled, and the breast-only bpCR endpoint (ypT0/is) did not incorporate post-treatment axillary nodal status. In addition, subgroup analyses were exploratory, and some strata contained relatively limited sample sizes; these findings should therefore be interpreted as supportive evidence of performance consistency rather than confirmatory subgroup-specific effects. Finally, all three centers were located within the same geographic region, limiting geographic and population heterogeneity. Further validation across broader geographic regions, diverse imaging platforms, and prospective clinical settings is needed to establish transportability and real-world clinical utility.

## 5. Conclusions

Pretreatment tri-modal deep learning integrating 2DUS, CDFI, and CEUS provided incremental predictive value for bpCR beyond the best-performing dual-modality model, with a consistent direction of benefit across two independent external centers. Further integration of pretreatment clinical and biomarker variables provided additional predictive information. These findings warrant further external and prospective evaluation of tri-modal ultrasound for individualized pretreatment bpCR prediction.

## Figures and Tables

**Figure 1:**
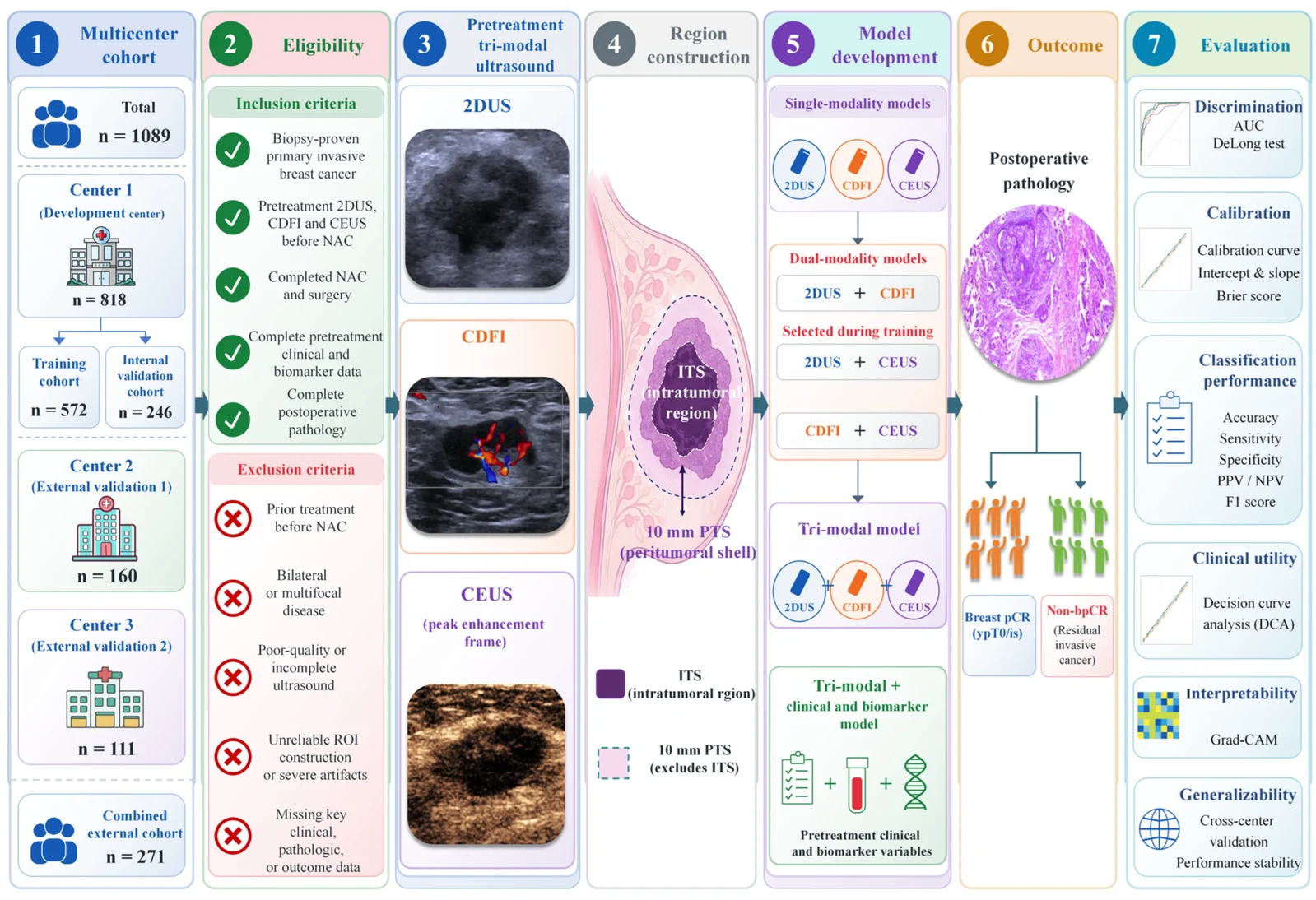
Multicenter study workflow illustrating patient selection, cohort allocation, pretreatment tri-modal ultrasound acquisition, intratumoral–peritumoral region construction, model development, pathologic outcome definition, and performance evaluation.

**Figure 3:**
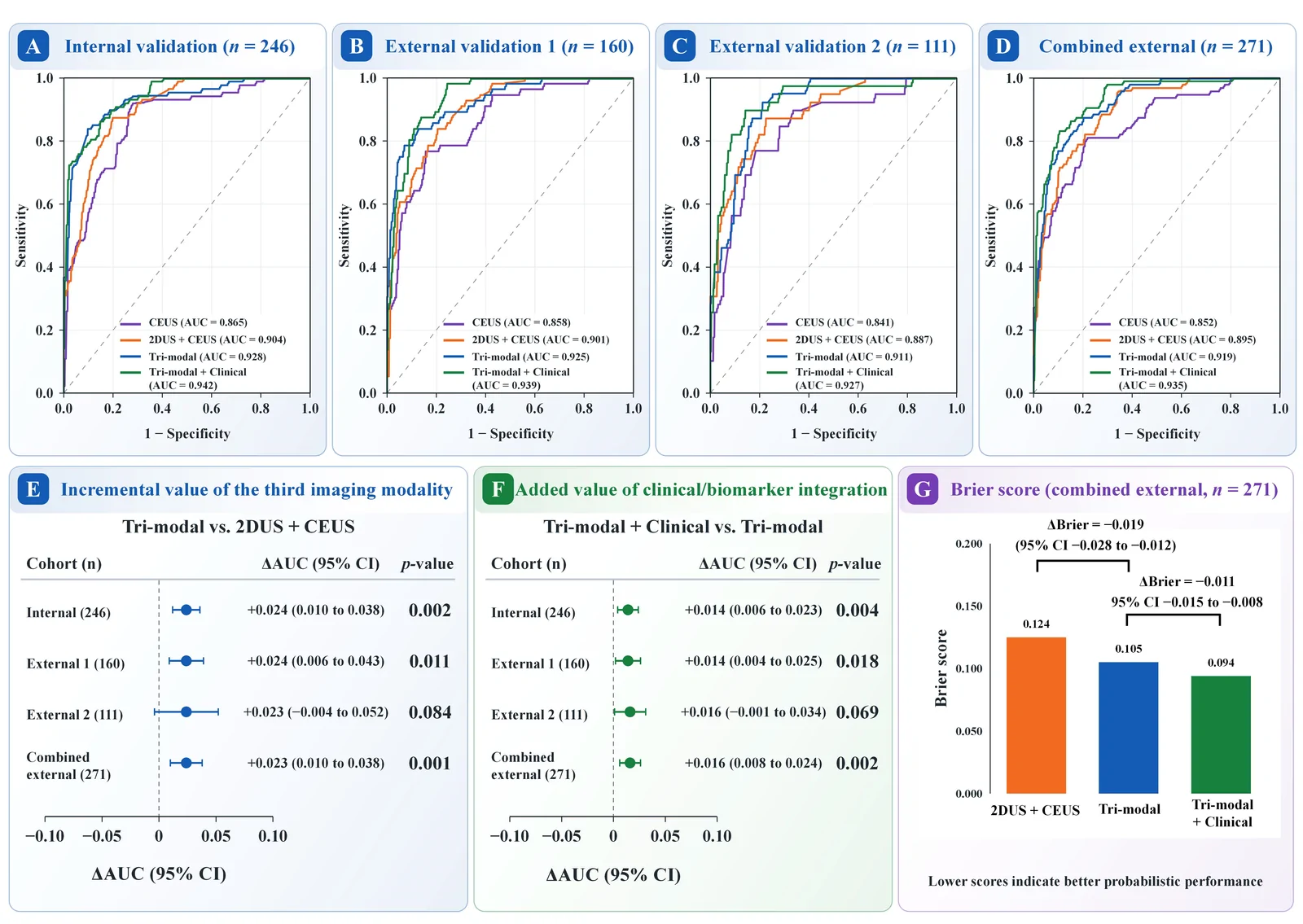
Comparative discrimination, incremental predictive value, and probabilistic performance of the best dual-modality, Tri-modal, and Tri-modal + Clinical models. (**A**–**D**) Receiver operating characteristic curves in the internal validation cohort, external validation cohort 1, external validation cohort 2, and combined external validation cohort, respectively. (**E**) Incremental value of adding CDFI to the locked 2DUS + CEUS model. (**F**) Additional value of integrating clinical and biomarker variables with the Tri-modal model. (**G**) Comparison of Brier scores among the 2DUS + CEUS, Tri-modal, and Tri-modal + Clinical models in the combined external validation cohort. Dashed diagonal lines in panels (**A**–**D**) indicate the no-discrimination reference, and dashed vertical lines in panels (**E**,**F**) indicate the null ΔAUC reference at 0.

**Figure 4:**
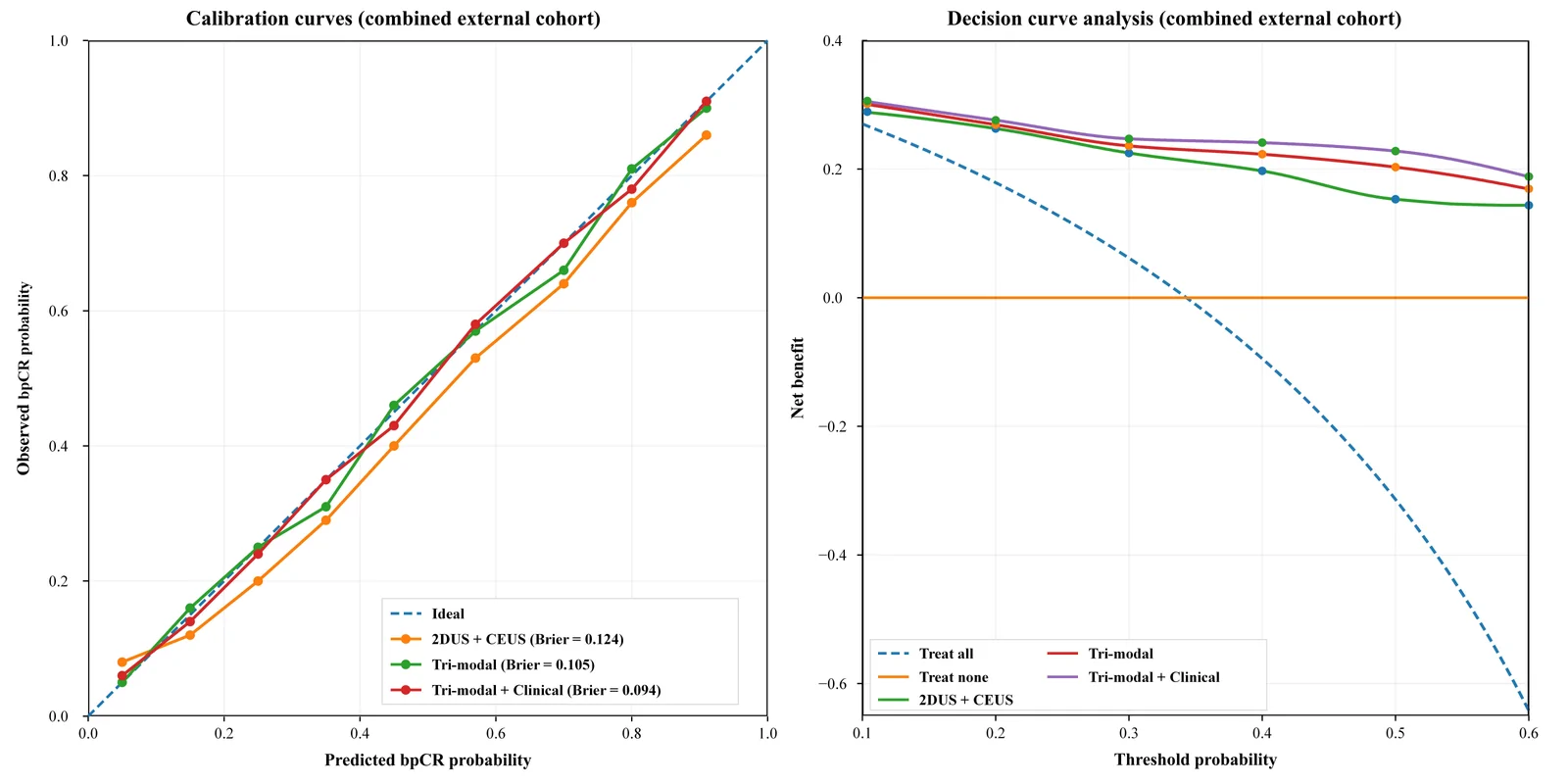
Calibration and decision curve analyses of the 2DUS + CEUS, Tri-modal, and Tri-modal + Clinical models in the combined external validation cohort. Colored circles are plotting markers for the corresponding model curves and have no additional categorical meaning.

**Figure 5:**
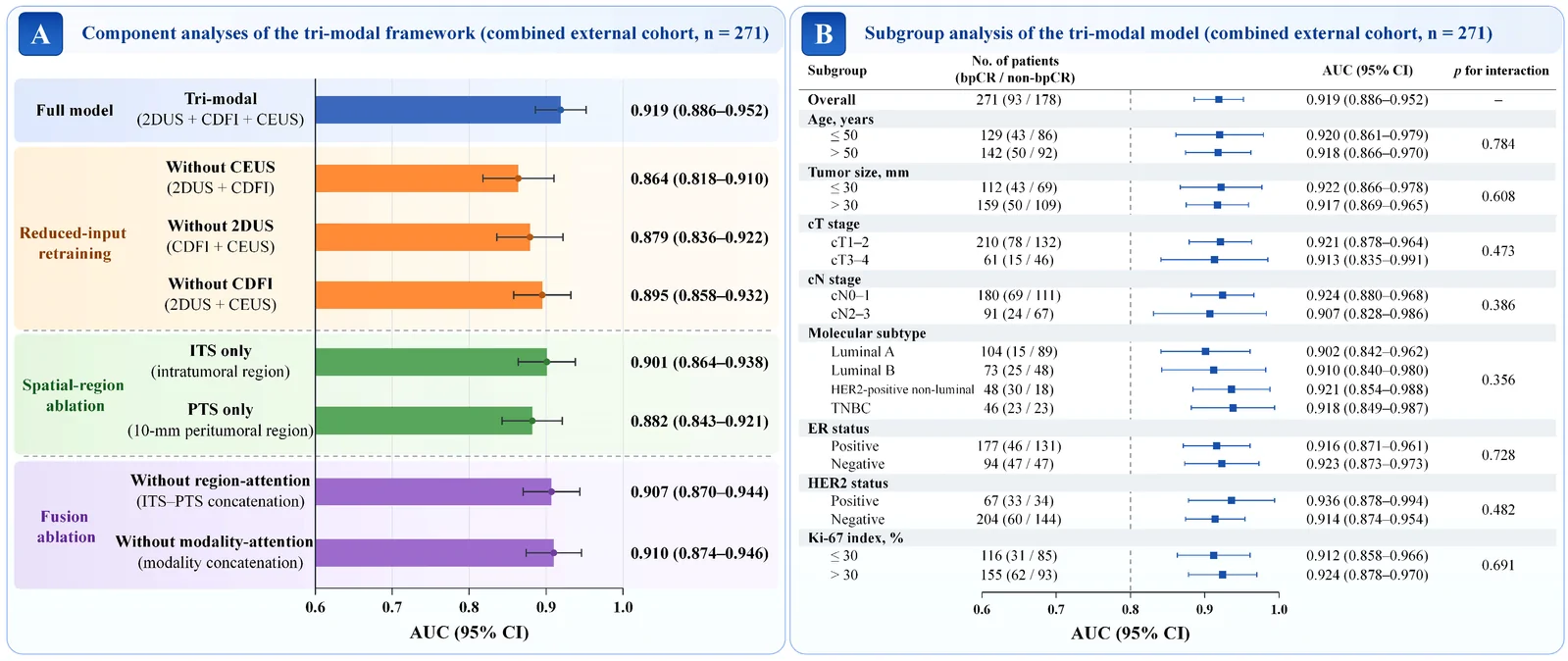
Component analyses and exploratory subgroup analyses evaluating model contributions and performance consistency in the combined external validation cohort. (**A**) Component and ablation analyses of the tri-modal framework, including reduced-input retraining, spatial-region ablation, and fusion ablation. Blue indicates the full model, orange indicates reduced-input retraining, green indicates spatial-region ablation, and purple indicates fusion ablation. (**B**) Exploratory subgroup analysis of the Tri-modal model across clinical and molecular subgroups; squares indicate subgroup-specific AUC estimates and horizontal lines indicate 95% confidence intervals.

**Figure 6:**
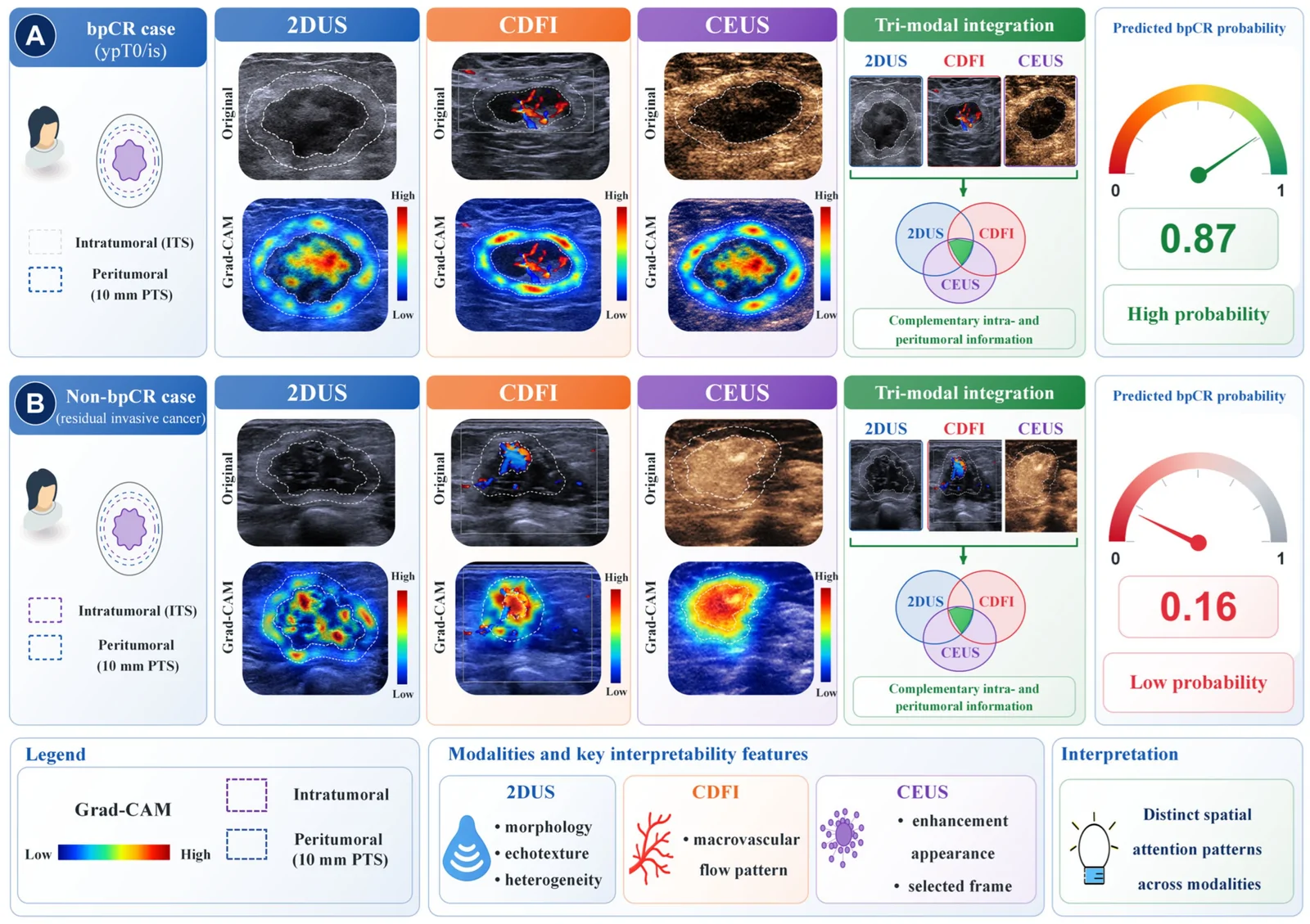
Representative Grad-CAM visualizations showing modality-specific spatial attention patterns in bpCR and non-bpCR cases.

**Table 1:** Baseline clinicopathologic characteristics across study cohorts.

Variable	Category	Overall (*n* = 1089)	Train (*n* = 572)	Internal Validation (*n* = 246)	External Validation 1 (*n* = 160)	External Validation 2 (*n* = 111)	*p*-Value
Age, years		52.7 ± 10.8	52.2 ± 10.7	53.0 ± 10.5	52.0 ± 11.0	54.1 ± 11.1	0.174
Baseline tumor size, mm		38.9 (30.6–47.2)	37.9 (30.0–46.1)	39.2 (31.0–47.6)	39.8 (31.4–48.1)	41.1 (32.0–49.2)	0.067
Ki-67, %		36.0 (15.0–58.0)	35.0 (14.0–56.0)	36.0 (15.0–58.0)	39.0 (16.0–60.0)	40.0 (16.0–62.0)	0.083
Molecular subtype	Luminal A	462 (42.4%)	252 (44.1%)	106 (43.1%)	64 (40.0%)	40 (36.0%)	0.537
	Luminal B	313 (28.7%)	168 (29.4%)	72 (29.3%)	45 (28.1%)	28 (25.2%)	
	HER2-positive non-luminal	163 (15.0%)	79 (13.8%)	36 (14.6%)	27 (16.9%)	21 (18.9%)	
	TNBC	151 (13.9%)	73 (12.8%)	32 (13.0%)	24 (15.0%)	22 (19.8%)	
cT stage	cT1	57 (5.2%)	38 (6.6%)	12 (4.9%)	5 (3.1%)	2 (1.8%)	0.041
	cT2	847 (77.8%)	450 (78.7%)	194 (78.9%)	125 (78.1%)	78 (70.3%)	
	cT3	176 (16.2%)	80 (14.0%)	38 (15.4%)	28 (17.5%)	30 (27.0%)	
	cT4	9 (0.8%)	4 (0.7%)	2 (0.8%)	2 (1.3%)	1 (0.9%)	
cN stage	cN0	308 (28.3%)	174 (30.4%)	67 (27.2%)	41 (25.6%)	26 (23.4%)	0.459
	cN1	457 (42.0%)	235 (41.1%)	109 (44.3%)	69 (43.1%)	44 (39.6%)	
	cN2	238 (21.9%)	122 (21.3%)	54 (22.0%)	36 (22.5%)	26 (23.4%)	
	cN3	86 (7.9%)	41 (7.2%)	16 (6.5%)	14 (8.8%)	15 (13.5%)	
ER status	Negative	314 (28.8%)	152 (26.6%)	68 (27.6%)	51 (31.9%)	43 (38.7%)	0.054
	Positive	775 (71.2%)	420 (73.4%)	178 (72.4%)	109 (68.1%)	68 (61.3%)	
PR status	Negative	430 (39.5%)	240 (42.0%)	89 (36.2%)	56 (35.0%)	45 (40.5%)	0.263
	Positive	659 (60.5%)	332 (58.0%)	157 (63.8%)	104 (65.0%)	66 (59.5%)	
HER2 status	Negative	858 (78.8%)	459 (80.2%)	195 (79.3%)	122 (76.3%)	82 (73.9%)	0.395
	Positive	231 (21.2%)	113 (19.8%)	51 (20.7%)	38 (23.8%)	29 (26.1%)	
Breast pathologic response	bpCR (ypT0/is)	383 (35.2%)	201 (35.1%)	89 (36.2%)	58 (36.3%)	35 (31.5%)	0.841
	non-bpCR	706 (64.8%)	371 (64.9%)	157 (63.8%)	102 (63.8%)	76 (68.5%)	

Data are mean ± SD, median (IQR), or n (%), as appropriate. *p*-values compare the four primary study cohorts. The HER2-positive non-luminal category refers to the molecular-subtype classification; HER2 status is reported separately.

**Table 2:** Performance hierarchy of Clinical/Biomarker, single-, dual-, and tri-modal models across validation cohorts.

Model	Internal AUC (95% CI)	Internal Brier	External 1 AUC (95% CI)	External 1 Brier	External 2 AUC (95% CI)	External 2 Brier	Combined External AUC (95% CI)	Combined External Brier
Clinical/ Biomarker	0.827 (0.772–0.882)	0.161	0.812 (0.742–0.882)	0.174	0.798 (0.714–0.882)	0.166	0.805 (0.750–0.860)	0.171
2DUS	0.836 (0.784–0.888)	0.154	0.829 (0.760–0.898)	0.157	0.814 (0.733–0.895)	0.168	0.823 (0.770–0.876)	0.162
CDFI	0.775 (0.715–0.835)	0.184	0.770 (0.691–0.849)	0.182	0.755 (0.661–0.849)	0.186	0.765 (0.705–0.825)	0.184
CEUS	0.865 (0.819–0.911)	0.143	0.858 (0.800–0.916)	0.146	0.841 (0.765–0.917)	0.154	0.852 (0.806–0.898)	0.149
2DUS + CDFI	0.877 (0.834–0.920)	0.136	0.871 (0.815–0.927)	0.134	0.853 (0.781–0.925)	0.145	0.864 (0.818–0.910)	0.139
2DUS + CEUS	0.904 (0.864–0.944)	0.118	0.901 (0.852–0.950)	0.121	0.887 (0.823–0.951)	0.128	0.895 (0.858–0.932)	0.124
CDFI + CEUS	0.890 (0.848–0.932)	0.124	0.884 (0.830–0.938)	0.127	0.870 (0.800–0.940)	0.132	0.879 (0.836–0.922)	0.129
Tri-modal	0.928 (0.895–0.961)	0.101	0.925 (0.884–0.966)	0.103	0.911 (0.853–0.969)	0.110	0.919 (0.886–0.952)	0.105
Tri-modal + Clinical	0.942 (0.913–0.971)	0.089	0.939 (0.904–0.974)	0.092	0.927 (0.876–0.978)	0.097	0.935 (0.906–0.964)	0.094

2DUS + CEUS was identified during training as the best dual-modality model and was locked as the primary comparator.

**Table 3:** Detailed discrimination, classification, probabilistic, and calibration performance of key models.

Dataset	Model	AUC (95% CI)	Locked Threshold	Accuracy	Sensitivity	Specificity	PPV	NPV	F1	Brier	Cal. Intercept	Cal. Slope
Internal validation	Clinical/ Biomarker	0.827 (0.772–0.882)	0.360	74.0%	71.9%	75.2%	62.1%	82.5%	0.667	0.161	−0.030	0.930
Internal validation	2DUS + CEUS	0.904 (0.864–0.944)	0.350	84.6%	82.0%	86.0%	76.8%	89.4%	0.793	0.118	0.080	1.060
Internal validation	Tri-modal	0.928 (0.895–0.961)	0.390	86.2%	84.3%	87.3%	78.9%	90.7%	0.815	0.101	0.070	1.030
Internal validation	Tri-modal + Clinical	0.942 (0.913–0.971)	0.370	88.2%	86.5%	89.2%	81.9%	92.1%	0.842	0.089	0.050	1.020
External validation 1	Clinical/ Biomarker	0.812 (0.742–0.882)	0.360	73.8%	72.4%	74.5%	61.8%	82.6%	0.667	0.174	−0.050	0.820
External validation 1	2DUS + CEUS	0.901 (0.852–0.950)	0.350	82.5%	81.0%	83.3%	73.4%	88.5%	0.770	0.121	0.110	1.040
External validation 1	Tri-modal	0.925 (0.884–0.966)	0.390	85.6%	84.5%	86.3%	77.8%	90.7%	0.810	0.103	0.100	1.020
External validation 1	Tri-modal + Clinical	0.939 (0.904–0.974)	0.370	87.5%	86.2%	88.2%	80.6%	91.8%	0.833	0.092	0.080	1.010
External validation 2	Clinical/ Biomarker	0.798 (0.714–0.882)	0.360	72.1%	68.6%	73.7%	54.5%	83.6%	0.608	0.166	0.030	0.880
External validation 2	2DUS + CEUS	0.887 (0.823–0.951)	0.350	80.2%	77.1%	81.6%	65.9%	88.6%	0.711	0.128	0.040	0.970
External validation 2	Tri-modal	0.911 (0.853–0.969)	0.390	82.9%	80.0%	84.2%	70.0%	90.1%	0.747	0.110	0.030	0.990
External validation 2	Tri-modal + Clinical	0.927 (0.876–0.978)	0.370	84.7%	82.9%	85.5%	72.5%	91.5%	0.773	0.097	0.020	1.000
Combined external	Clinical/ Biomarker	0.805 (0.750–0.860)	0.360	73.1%	71.0%	74.2%	58.9%	83.0%	0.644	0.171	−0.020	0.850
Combined external	2DUS + CEUS	0.895 (0.858–0.932)	0.350	81.5%	79.6%	82.6%	70.5%	88.6%	0.747	0.124	0.080	1.010
Combined external	Tri-modal	0.919 (0.886–0.952)	0.390	84.5%	82.8%	85.4%	74.8%	90.5%	0.786	0.105	0.080	1.010
Combined external	Tri-modal + Clinical	0.935 (0.906–0.964)	0.370	86.3%	84.9%	87.1%	77.5%	91.7%	0.810	0.094	0.060	1.000

Classification thresholds were fixed from training-stage out-of-fold predictions. Calibration intercepts close to 0 and slopes close to 1 indicate better calibration.

**Table 4:** Incremental predictive value of tri-modal ultrasound and clinical/biomarker integration.

Dataset	Analysis	Comparison	AUC (Reference/Comparison)	ΔAUC (95% CI)	DeLong *p*	ΔAccuracy (95% CI)	ΔF1 (95% CI)	ΔBrier (95% CI)	Relative Brier Reduction
Internal validation	Primary	2DUS + CEUS vs. Tri-modal	0.904/0.928	+0.024 (+0.010 to +0.038)	0.002	+0.016 (−0.010 to +0.042)	+0.022 (−0.012 to +0.057)	−0.017 (−0.024 to −0.010)	14.4%
External validation 1	Primary	2DUS + CEUS vs. Tri-modal	0.901/0.925	+0.024 (+0.006 to +0.043)	0.011	+0.031 (+0.002 to +0.067)	+0.040 (+0.003 to +0.081)	−0.018 (−0.028 to −0.009)	14.9%
External validation 2	Primary	2DUS + CEUS vs. Tri-modal	0.887/0.911	+0.023 (−0.004 to +0.052)	0.084	+0.027 (−0.009 to +0.072)	+0.036 (−0.008 to +0.081)	−0.018 (−0.033 to −0.005)	14.1%
Combined external	Primary	2DUS + CEUS vs. Tri-modal	0.895/0.919	+0.023 (+0.010 to +0.038)	0.001	+0.030 (+0.009 to +0.052)	+0.039 (+0.013 to +0.074)	−0.019 (−0.028 to −0.012)	15.3%
Internal validation	Secondary	Tri-modal vs. Tri-modal + Clinical	0.928/0.942	+0.014 (+0.006 to +0.023)	0.004	+0.020 (+0.001 to +0.043)	+0.027 (+0.004 to +0.054)	−0.012 (−0.016 to −0.008)	11.9%
External validation 1	Secondary	Tri-modal vs. Tri-modal + Clinical	0.925/0.939	+0.014 (+0.004 to +0.025)	0.018	+0.019 (−0.002 to +0.044)	+0.023 (−0.002 to +0.056)	−0.011 (−0.015 to −0.007)	10.7%
External validation 2	Secondary	Tri-modal vs. Tri-modal + Clinical	0.911/0.927	+0.016 (−0.001 to +0.034)	0.069	+0.018 (−0.011 to +0.050)	+0.026 (−0.010 to +0.060)	−0.013 (−0.019 to −0.007)	11.8%
Combined external	Secondary	Tri-modal vs. Tri-modal + Clinical	0.919/0.935	+0.016 (+0.008 to +0.024)	0.002	+0.018 (+0.003 to +0.037)	+0.024 (+0.005 to +0.054)	−0.011 (−0.015 to −0.008)	10.5%

Primary comparison: 2DUS + CEUS versus Tri-modal. Secondary comparison: Tri-modal versus Tri-modal + Clinical. Negative ΔBrier indicates improved probabilistic performance.

**Table 5:** Cross-center performance stability of the Tri-modal and Tri-modal + Clinical models.

Model	External Evaluation	Internal AUC	External AUC	ΔAUC vs. Internal	Internal Brier	External Brier	ΔBrier	External Cal. Intercept	External Cal. Slope	External F1
Tri-modal	External validation 1	0.928	0.925	−0.003	0.101	0.103	+0.002	0.100	1.020	0.810
Tri-modal	External validation 2	0.928	0.911	−0.017	0.101	0.110	+0.009	0.030	0.990	0.747
Tri-modal	Combined external	0.928	0.919	−0.009	0.101	0.105	+0.004	0.080	1.010	0.786
Tri-modal + Clinical	External validation 1	0.942	0.939	−0.003	0.089	0.092	+0.003	0.080	1.010	0.833
Tri-modal + Clinical	External validation 2	0.942	0.927	−0.015	0.089	0.097	+0.008	0.020	1.000	0.773
Tri-modal + Clinical	Combined external	0.942	0.935	−0.007	0.089	0.094	+0.005	0.060	1.000	0.810

External cohorts were evaluated without retraining, threshold adjustment, or center-specific recalibration. ΔAUC = external AUC − internal AUC; ΔBrier = external Brier − internal Brier.

**Table 6:** Architecture-matched control and ablation analyses of modality, spatial-region, and fusion components.

Analysis Category	Model/ Configuration	Internal AUC (Δ)	External 1 AUC (Δ)	External 2 AUC (Δ)	Combined External AUC (95% CI; Δ)
Full model	Full Tri-modal	0.928 (reference)	0.925 (reference)	0.911 (reference)	0.919 (0.886–0.952; reference)
Architecture-matched control	2DUS + CEUS + duplicated 2DUS	0.906 (−0.022)	0.903 (−0.022)	0.889 (−0.022)	0.898 (0.854–0.944; −0.021)
Reduced- input retraining	Without CEUS (2DUS + CDFI)	0.877 (−0.051)	0.871 (−0.054)	0.853 (−0.058)	0.864 (0.818–0.910; −0.055)
Reduced- input retraining	Without 2DUS (CDFI + CEUS)	0.890 (−0.038)	0.884 (−0.041)	0.870 (−0.041)	0.879 (0.836–0.922; −0.040)
Reduced- input retraining	Without CDFI (2DUS + CEUS)	0.904 (−0.024)	0.901 (−0.024)	0.887 (−0.023)	0.895 (0.858–0.932; −0.023)
Spatial ablation	ITS only (no PTS)	0.910 (−0.018)	0.906 (−0.019)	0.892 (−0.019)	0.901 (0.864–0.938; −0.018)
Spatial ablation	PTS only (no ITS)	0.892 (−0.036)	0.887 (−0.038)	0.873 (−0.038)	0.882 (0.843–0.921; −0.037)
Fusion ablation	Without region-attention (ITS–PTS concatenation)	0.916 (−0.012)	0.912 (−0.013)	0.899 (−0.012)	0.907 (0.870–0.944; −0.012)
Fusion ablation	Without modality-attention (modality concatenation)	0.918 (−0.010)	0.915 (−0.010)	0.902 (−0.009)	0.910 (0.874–0.946; −0.009)

The full Tri-modal model is the reference. The architecture-matched control replaced CDFI with duplicated 2DUS input. Reduced-input models were retrained after omitting one modality rather than being generated by inference-time occlusion of the trained Tri-modal model. Negative ΔAUC values indicate lower performance than the full model.

## Data Availability

The data presented in this study are available from the corresponding author upon reasonable request. The data are not publicly available because they contain information that could compromise patient privacy and are subject to institutional ethical restrictions.

## Supplementary Materials

The following supporting information can be downloaded at: https://www.mdpi.com/article/10.3390/cancers18203326/s1. Table S1: Clinicopathologic characteristics according to breast pathologic response; Table S2: ROI delineation repeatability and prediction-level robustness; Table S3: Locked training-stage thresholds and out-of-fold reference performance; Table S4: Stand-alone performance and incremental contribution of each ultrasound modality in the combined external cohort; Table S5: Decision-curve net benefit in the combined external cohort; Table S6: Exploratory subgroup performance of the Tri-modal model in the combined external cohort; Table S7: Exploratory molecular-subtype performance in the combined external cohort.
