# Supplementary material for: Incremental Value and Cross-Center Validation of Pretreatment Tri-Modal Ultrasound Deep Learning for Predicting Breast Pathologic Complete Response to Neoadjuvant Chemotherapy: A Multicenter Study
Source: Cancers (Basel). 2026 Oct 15;18(20):3326. doi: 10.3390/cancers18203326 (PMC13702602; doi:10.3390/cancers18203326)
Supplement: Supplementary file 1 [file cancers-18-03326-s001.zip › cancers-4632666-supplementary.pdf]

Supplementary Tables

Supplementary Table S1. Clinicopathologic characteristics according to breast pathologic response

Variable	Category	bpCR (n=383)	non-bpCR (n=706)	P value
Age, years		52.4 ± 10.6	52.9 ± 10.9	0.451
Baseline tumor size, mm		33.4 (26.6–40.9)	41.4 (33.7–49.5)	<0.001
Ki-67, %		48.0 (28.0–68.0)	32.0 (12.0–52.0)	<0.001
Molecular subtype	Luminal A	82 (21.4%)	380 (53.8%)	<0.001
	Luminal B	108 (28.2%)	205 (29.0%)	
	HER2-positive non-luminal	104 (27.2%)	59 (8.4%)	
	TNBC	89 (23.2%)	62 (8.8%)	
cT stage	cT1	27 (7.0%)	30 (4.2%)	<0.001
	cT2	322 (84.1%)	525 (74.4%)	
	cT3	33 (8.6%)	143 (20.3%)	
	cT4	1 (0.3%)	8 (1.1%)	
cN stage	cN0	130 (33.9%)	178 (25.2%)	<0.001
	cN1	165 (43.1%)	292 (41.4%)	
	cN2	67 (17.5%)	171 (24.2%)	
	cN3	21 (5.5%)	65 (9.2%)	
ER status	Negative	193 (50.4%)	121 (17.1%)	<0.001
	Positive	190 (49.6%)	585 (82.9%)	
PR status	Negative	213 (55.6%)	217 (30.7%)	<0.001
	Positive	170 (44.4%)	489 (69.3%)	
HER2 status	Negative	253 (66.1%)	605 (85.7%)	<0.001
	Positive	130 (33.9%)	101 (14.3%)	

Comparisons are descriptive and are not interpreted as independent causal associations.

Supplementary Table S2. ROI delineation repeatability and prediction-level robustness

Modality	Repeated cases	Dice, mean ± SD
2DUS	109	0.923 ± 0.019
CDFI	109	0.897 ± 0.027
CEUS	109	0.912 ± 0.023

Approximately 10% of the cohort (n=109) was independently re-delineated for repeatability assessment.

Prediction-level robustness analysis

Metric	Result
Predicted-probability ICC	0.946
Mean absolute probability difference	0.034
AUC, primary ROI	0.913
AUC, repeat ROI	0.905
Absolute AUC difference	0.008

A total of 109 randomly selected patients underwent independent repeat delineation, with geometric and prediction-level reproducibility assessed using the two ROI sets.

Supplementary Table S3. Locked training-stage thresholds and out-of-fold reference performance

Model	Locked threshold	Training out-of-fold AUC (95% CI)
Clinical/Biomarker	0.360	0.850 (0.819–0.881)
2DUS	0.440	0.842 (0.810–0.874)
CDFI	0.400	0.778 (0.739–0.817)
CEUS	0.350	0.872 (0.842–0.902)
2DUS+CDFI	0.410	0.883 (0.855–0.911)
2DUS+CEUS	0.350	0.913 (0.889–0.937)
CDFI+CEUS	0.360	0.894 (0.867–0.921)
Tri-modal	0.390	0.935 (0.915–0.955)
Tri-modal+Clinical	0.370	0.948 (0.930–0.966)

Thresholds were determined during model development and locked before independent validation.

Supplementary Table S4. Stand-alone performance and incremental contribution of each ultrasound modality in the combined external cohort

Modality	Stand-alone AUC	Model without modality	AUC without modality	Full Tri-modal AUC	AUC gain after adding modality
2DUS	0.823	CDFI+CEUS	0.879	0.919	+0.040
CDFI	0.765	2DUS+CEUS	0.895	0.919	+0.023
CEUS	0.852	2DUS+CDFI	0.864	0.919	+0.055

Values are rearranged from the reported single-/dual-modality and reduced-input retraining analyses. This descriptive table illustrates that stand-alone discrimination and incremental contribution are related but not identical.

Supplementary Table S5. Decision-curve net benefit in the combined external cohort

Threshold probability	Treat all	Treat none	Best dual	Tri-modal	Tri-modal+Clinical
0.10	0.270	0.000	0.289	0.302	0.306
0.20	0.179	0.000	0.263	0.269	0.276
0.30	0.062	0.000	0.225	0.236	0.247
0.40	-0.095	0.000	0.197	0.223	0.241
0.50	-0.314	0.000	0.153	0.203	0.228
0.60	-0.642	0.000	0.141	0.169	0.188

Net benefit should be interpreted across threshold ranges rather than at a single threshold.

Supplementary Table S6. Exploratory subgroup performance of the Tri-modal model in the combined external cohort

Subgroup	Category	N	bpCR, n (%)	non-bpCR, n (%)	Tri-modal AUC (95% CI)	*P* for interaction
Overall	—	271	93 (34.3%)	178 (65.7%)	0.919 (0.886–0.952)	—
Age, years	≤ 50	129	43 (33.3%)	86 (66.7%)	0.920 (0.861–0.979)	0.784
	> 50	142	50 (35.2%)	92 (64.8%)	0.918 (0.866–0.970)	
Tumor size, mm	≤ 30	112	43 (38.4%)	69 (61.6%)	0.922 (0.866–0.978)	0.608
	> 30	159	50 (31.4%)	109 (68.6%)	0.917 (0.869–0.965)	
cT stage	cT1–2	210	78 (37.1%)	132 (62.9%)	0.921 (0.878–0.964)	0.473

Subgroup	Category	N	bpCR, n (%)	non-bpCR, n (%)	Tri-modal AUC (95% CI)	*P* for interaction
	cT3–4	61	15 (24.6%)	46 (75.4%)	0.913 (0.835–0.991)	
cN stage	cN0–1	180	69 (38.3%)	111 (61.7%)	0.924 (0.880–0.968)	0.386
	cN2–3	91	24 (26.4%)	67 (73.6%)	0.907 (0.828–0.986)	
Molecular subtype	Luminal A	104	15 (14.4%)	89 (85.6%)	0.902 (0.842–0.962)	0.356
	Luminal B	73	25 (34.2%)	48 (65.8%)	0.910 (0.840–0.980)	
	HER2-positive non-luminal	48	30 (62.5%)	18 (37.5%)	0.921 (0.854–0.988)	
	TNBC	46	23 (50.0%)	23 (50.0%)	0.918 (0.849–0.987)	
ER status	Positive	177	46 (26.0%)	131 (74.0%)	0.916 (0.871–0.961)	0.728
	Negative	94	47 (50.0%)	47 (50.0%)	0.923 (0.873–0.973)	
HER2 status	Positive	67	33 (49.3%)	34 (50.7%)	0.936 (0.878–0.994)	0.482
	Negative	204	60 (29.4%)	144 (70.6%)	0.914 (0.874–0.954)	
Ki-67 index, %	≤ 30	116	31 (26.7%)	85 (73.3%)	0.912 (0.858–0.966)	0.691
	> 30	155	62 (40.0%)	93 (60.0%)	0.924 (0.878–0.970)	

AUCs are for the Tri-modal model in the combined external cohort (n=271; 93 bpCR and 178 non-bpCR). Subgroup analyses are exploratory. P values for interaction were obtained from likelihood-ratio tests of the interaction between the continuous Tri-modal prediction score and subgroup variables; interaction terms for multi-level subgroup variables were tested jointly.

Supplementary Table S7. Exploratory molecular-subtype performance in the combined external cohort

Subtype	N	bpCR, n (%)	Best dual AUC	Tri-modal AUC	ΔAUC	Tri-modal+Clinical AUC
Luminal A	104	15 (14.4%)	0.879	0.902	+0.023	0.919
Luminal B	73	25 (34.2%)	0.886	0.910	+0.024	0.928
HER2-positive non-luminal	48	30 (62.5%)	0.895	0.921	+0.026	0.938
TNBC	46	23 (50.0%)	0.882	0.918	+0.036	0.934

Subtype analyses are exploratory because of limited subgroup sample sizes; no subtype-specific model claim is made.
